# Cardiovascular risk factors and metabolic syndrome in people with established psychotic illnesses: baseline data from the IMPaCT randomized controlled trial

**DOI:** 10.1017/S0033291715000562

**Published:** 2015-05-12

**Authors:** P. Gardner-Sood, J. Lally, S. Smith, Z. Atakan, K. Ismail, K. E. Greenwood, A. Keen, C. O'Brien, O. Onagbesan, C. Fung, E. Papanastasiou, J. Eberherd, A. Patel, R. Ohlsen, D. Stahl, A. David, D. Hopkins, R. M. Murray, F. Gaughran, on behalf of the IMPaCT team

**Affiliations:** 1 Department of Psychosis Studies, Institute of Psychiatry, Psychology and Neuroscience (IoPPN), King's College London, London, UK; 2 National Psychosis Service, South London and Maudsley NHS Foundation Trust, London, UK; 3 Institute of Psychiatry, Psychology and Neuroscience (IoPPN), King's College London, London, UK; 4 South London and Maudsley NHS Foundation Trust, London, UK; 5 King's College Hospital NHS Foundation Trust, London, UK; 6 School of Psychology, University of Sussex, Brighton and Early Intervention in Psychosis Service, Sussex Partnership NHS Foundation Trust, West Sussex, UK; 7 NIHR Biomedical Research Centre – BioResource for Mental Health, Social, Genetic and Development Psychiatric Centre, London, UK; 8 Clinical Sciences, Lund University, Malmö, Sweden; 9 Centre for the Economics of Mental and Physical Health (CEMPH), Institute of Psychiatry, Psychology and Neuroscience (IoPPN), King's College London, London, UK; 10 Florence Nightingale Faculty of Nursing and Midwifery, King's College London, London, UK; 11 Department of Biostatistics, Institute of Psychiatry, Psychology and Neuroscience (IoPPN), King's College London, London, UK; 12 Division of Ambulatory Care and Local Networks, King's College Hospital NHS Foundation Trust, London, UK; 13 King's College London School of Medicine, London, UK; 14 Institute of Psychiatry, Psychology and Neuroscience (IoPPN) and the Biomedical Research Centre, BRC Nucleus, Maudsley Hospital, South London and Maudsley NHS Foundation Trust, Denmark Hill, London, UK

**Keywords:** Metabolic syndrome, physical health, psychotic disorder, schizophrenia, severe mental illnesses

## Abstract

**Background:**

The aims of the study were to determine the prevalence of cardiometabolic risk factors and establish the proportion of people with psychosis meeting criteria for the metabolic syndrome (MetS). The study also aimed to identify the key lifestyle behaviours associated with increased risk of the MetS and to investigate whether the MetS is associated with illness severity and degree of functional impairment.

**Method:**

Baseline data were collected as part of a large randomized controlled trial (IMPaCT RCT). The study took place within community mental health teams in five Mental Health NHS Trusts in urban and rural locations across England. A total of 450 randomly selected out-patients, aged 18–65 years, with an established psychotic illness were recruited. We ascertained the prevalence rates of cardiometabolic risk factors, illness severity and functional impairment and calculated rates of the MetS, using International Diabetes Federation (IDF) and National Cholesterol Education Program Third Adult Treatment Panel criteria.

**Results:**

High rates of cardiometabolic risk factors were found. Nearly all women and most men had waist circumference exceeding the IDF threshold for central obesity. Half the sample was obese (body mass index ≥ 30 kg/m^2^) and a fifth met the criteria for type 2 diabetes mellitus. Females were more likely to be obese than males (61% *v.* 42%, *p* < 0.001). Of the 308 patients with complete laboratory measures, 57% (*n* = 175) met the IDF criteria for the MetS.

**Conclusions:**

In the UK, the prevalence of cardiometabolic risk factors in individuals with psychotic illnesses is much higher than that observed in national general population studies as well as in most international studies of patients with psychosis.

## Introduction

The diagnosis of a psychotic illness such as schizophrenia or bipolar disorder is associated with a reduced life expectancy of 15–25 years, mostly due to increased cardiovascular deaths (Saha *et al.* 2007; Kilbourne *et al.* 2009; Brown *et al.* 2010; Chang *et al.* 2011). A recent meta-analysis has demonstrated that the pooled relative risk of mortality among those with psychoses is 2.54 (2.35–2.75) times that of the general population, and higher rates of mortality for those with psychoses when compared with depressive and anxiety disorders (Walker *et al.* 2015). Cardiometabolic risk factors are common in psychosis (McEvoy *et al.* 2005; Leucht *et al.* 2007; Osborn *et al.* 2007; De Hert *et al.* 2009; Kilbourne *et al.* 2009). A recent meta-analysis of international studies reported prevalence rates of 44% for central obesity, 19.5% for hyperglycaemia, 10.9% for diabetes, 39% for hypertension and 39% for dyslipidaemias, with rates increasing with age (Mitchell *et al.* 2013).

Clustering of cardiometabolic risk factors is termed the metabolic syndrome (MetS) (Alberti *et al.* 2005). Individuals with the MetS have a 3- to 6-fold increased risk of developing type 2 diabetes mellitus (Hanley *et al.* 2005; De Hert *et al.* 2011) and a 2- to 6-fold risk of mortality due to cardiovascular disease (CVD) (Hanley *et al.* 2005). On meta-analysis, a third of patients with schizophrenia have the MetS, with this proportion increasing with duration of illness (Mitchell *et al.* 2013). Slightly higher MetS rates of 34% are seen in multi-episode patients (Vancampfort *et al.* 2013*b*) while the deficit syndrome (i.e. negative symptoms of psychosis that are present as enduring traits) in schizophrenia is in itself associated with higher CVD risk (Arango *et al.* 2011). Similar rates of the MetS have been observed in bipolar disorder (37%) (Vancampfort *et al.* 2013*a*) and schizo-affective disorder (27–42%) (Basu *et al.* 2004; Bobes *et al.* 2012), although recently the Second Australian National Survey of Psychosis reported higher rates of 61% in those with psychotic disorders, suggesting an increasing prevalence with time (Morgan *et al.* 2014). It is possible to prevent the emergence of CVD risk in psychosis with the use of psychosocial interventions in the short term, though this is not sustained once the intervention is discontinued (Alvarez-Jimenez *et al.* 2010). Strategies do exist to manage established cardiometabolic risk factors (Lester *et al.* 2014), but despite this, a recent UK national audit reported screening rates for metabolic side effects of antipsychotic medication of only 11% (Barnes *et al.* 2007). This is further compounded by the low treatment rates of identified cardiometabolic risk factors in psychosis (Nasrallah *et al.* 2006). The recent Schizophrenia Commission Report (UK) highlighted the disparity of care for individuals with psychosis and emphasized the need for improved physical health intervention (Schizophrenia Commission, 2012). Encouragingly, various UK medical Royal colleges have recently agreed joint management strategies for CVD risk in psychotic illnesses (Lester *et al.* 2014).

In this study we aimed:

(1) To determine the prevalence of cardiometabolic risks factors and establish the proportion of people with psychosis meeting the International Diabetes Federation (IDF) criteria for the MetS.
(2) To identify the key lifestyle behaviours associated with increased risk of the MetS.
(3) To investigate whether the presence of the MetS is associated with illness severity and degree of functional impairment.

## Method

### Study design

This study used baseline data from 450 randomly selected patients with established (multi-episode) psychosis recruited as part of the National Institute for Health Research-funded study: Improving Physical Health and Reducing Substance Use in Severe Mental Illness; a randomized controlled trial (IMPaCT RCT) (Gaughran *et al.* 2013).

#### Setting

The study took place in community mental health teams (CMHTs) in five Mental Health NHS Trusts, covering urban (Lambeth, Southwark, Lewisham, Croydon, Greenwich, Bexley, Bromley) and rural (Staffordshire, Somerset and Sussex) boroughs across England (Gaughran *et al*. 2013).

#### Subjects

Potential participants were identified through their care-coordinator. All care-coordinators in participating CMHTs were approached in a random sequence and invited to participate. Patients on each participating care coordinator's caseload, meeting the inclusion criteria, were likewise approached in a random order. These patients were entered into a random numbers generator to create a randomly ordered list from which to approach potential participants. Researchers then approached these patients sequentially to participate in the RCT. In situations where a patient did not wish to take part in the study or was uncontactable, the researcher selected another patient from the list in that random order.

The inclusion criteria were as follows: aged between 18 and 65 years old; a primary diagnosis of a psychotic illness [International Classification of Diseases (ICD)-10 diagnosis: F20–29, including schizophrenia, schizo-affective disorder, bipolar affective disorder (BPAD) and delusional disorder, F31.2, F32.3 and F33.3]. The exclusion criteria included: a primary diagnosis of intellectual disability (as defined by ICD-10 codes F70–F79 for intellectual disabilities); a first episode of psychosis (FEP); a primary substance misuse disorder (excluding cigarettes); a serious physical illness that could independently have an impact on metabolic measures; pregnant or up to 6 months postpartum; and receiving intensive input for a medical or terminal condition. Of patients screened, 39% (1043/2663) met the inclusion criteria, with the majority excluded for not meeting the diagnostic inclusion criterion (though the exact percentage excluded by each exclusion criterion is not known).

### Clinical and sociodemographic variables

Sociodemographic and clinical data including gender, age, ethnicity, diagnosis and self-reported duration of illness were collected. Diagnoses were based on ICD-10 diagnostic criteria and were extracted from the documented diagnosis in the clinical notes at the time of recruitment. Participants’ mental health status was measured using the Positive And Negative Syndrome Scale (PANSS; Kay *et al.* 1989), the Global Assessment of Functioning (GAF; American Psychiatric Association, 2002) and the Montgomery–Åsberg Depression Rating Scale (MADRS; Montgomery & Åsberg, 1979). Current alcohol, smoking and cannabis use was recorded using the following measures: the Alcohol Use Disorders Identification Test (AUDIT; Saunders *et al*. 1993); the Nicotine Dependence Questionnaire (Fagerstrom, 1978; Fagerstrom & Schneider, 1989); Timeline Follow-Back (Sobell & Sobell, 1992); and a urine drug screen. Total scores were derived from individual scale items. If any individual items were missing, then the total score was treated as missing. Data for all measures were collected through face-to-face interviews conducted by trained research assistants.

### Cardiometabolic risk factors

Height, weight and blood pressure (BP) were measured using standardized techniques. The BP values presented are the second BP measurements, taken after an interval of 5 min. Waist circumference was measured at the umbilicus with the patient standing. As both serum glucose and triglycerides are affected by recent food ingestion, we took fasting blood samples.

Obesity was defined using the World Health Organization reference standard [body mass index (BMI) ≥ 30 kg/m^2^]. Diabetes was diagnosed based on measured glycated haemoglobin (HbA1c) of ≥48 mmol/mol (≥6.5%) or fasting glucose ≥7.0 mmol/l or a prior diagnosis of diabetes. In addition, in keeping with recent guidance, an HbA1c of 42–46 mmol/mol (6–6.4%) was taken to indicate glucose dysregulation or a high risk of diabetes.

The IDF definitions of other cardiometabolic risk factors and of the MetS were used (see Table 1) (Alberti *et al.* 2005, 2006). However, we extended the definition of the MetS to include an HbA1c value ≥42 mmol/mol (≥6%), as this is consistent with a raised fasting plasma glucose ≥5.6 mmol/l (The International Expert Committee, 2009; Lester *et al.* 2014).

**Table 1.** Diagnostic criteria for the metabolic syndrome (International Diabetes Federation criteria) (Alberti et al. 2006)

Diagnostic criteria	
Central obesity	
Men	Waist circumference ≥94 cm in Europid men, ≥90 cm in South Asian men
Women	Waist circumference ≥80 cm in Europid women, ≥80 cm in South Asian women
Plus any two of the following: raised triglycerides	≥1.7 mmol/l or specific treatment for this lipid abnormality
High-density lipoprotein-cholesterol	
Men	<1.03 mmol/l
Women	<1.29 mmol/l or specific treatment for this lipid abnormality
Raised blood pressure	Systolic blood pressure ≥130 mmHg or diastolic blood pressure ≥85 mmHg or treatment of previously diagnosed hypertension
Raised fasting plasma glucose	≥5.6 mmol/l or previously diagnosed type 2 diabetes

Additionally, we also determined if each patient met the adapted National Cholesterol Education Program (NCEP) Third Adult Treatment Panel (ATP-III) criteria (NCEP ATP-III criteria) (Grundy *et al.* 2005), to allow for comparison with selected studies. The ATP-III classification requires three of five criteria to be met for a diagnosis of the MetS and an abnormal waist circumference is not obligatory. In comparison with the IDF MetS criteria, the ATP-III criteria have a higher threshold for abnormal waist circumference, with 102 cm for males and 88 cm for females being the cut-offs. The other four criteria are comparable with the IDF criteria.

Hypertension was defined on the basis of a measured BP of either a systolic measure of 130 mmHg and/or a diastolic measure of 85 mmHg or existing treatment with antihypertensive medication.

The Dietary Instrument for Nutrition Education (DINE; Roe *et al.* 1994) was used to assess the dietary patterns of participants over the past week. Respondents were classified according to their saturated fat, unsaturated fat and fibre intake, based on self-reported consumption of various foods. The International Physical Activity Questionnaire was used to measure the intensity of physical activity (high, moderate, low) and the duration of physical activities over the previous week (Craig *et al.* 2003).

### Statistical analysis

Statistical analyses were performed using the IBM Statistical Package for Social Sciences Statistics for Windows, version 20.0 (USA). Descriptive measures were used for the basic demographic and clinical variables as well as for variables relating to the evaluation of metabolic dysregulation. Student's *t* test for parametric data and the *χ*^2^ test for categorical variables were employed. All statistical tests were two-sided and a *p* value ≤0.05 was considered statistically significant.

### Ethical Standards

Ethical approval for this study was obtained from The Joint South London and Maudsley and The Institute of Psychiatry NHS Research Ethics Committee. Ethical approval was granted on 17 July 2009 (REC ref. no. 09/H080/41).

## Results

The clinical characteristics of the study population are shown in Table 2. Mean age was 43.6 years (s.d. = 10.1) and mean duration of illness was 15.7 years (s.d. = 10.3); 57% (*n* = 257) were male. In respect of ethnicity, 55% (*n* = 239) were of Caucasian ethnicity, while 33% (*n* = 143) were of black African or black Caribbean ethnicity, with 7% (*n* = 27) of Asian ethnicity and 5% (*n* = 22) of mixed ethnicity.

**Table 2.** Clinical characteristics of the study population (n = 450)

	Total	Males (*n* = 257)	Females (*n* = 193)
Mean age, years (s.d.) (*n* = 449)	43.6 (10.1)	42.7 (9.9)	44.8 (10.2)
Mean duration of illness, years (s.d.) (*n* = 358)	15.7 (10.3)	16.5 (10.3)	14.7 (10.3)
Mean PANSS total score (s.d.) (*n* = 417)	51.2 (14.1)	51.5 (14.3)	50.9 (13.9)
Mean PANSS positive symptom scale score (s.d.) (*n* = 421)	11.8 (5.0)	12.0 (5.1)	11.5 (4.8)
Mean PANSS negative symptom scale score (s.d.) (*n* = 418)	12.9 (4.9)	13.1 (4.9)	12.6 (5.0)
Mean MADRS score (s.d.) (*n* = 427)	11.0 (9.5)	10.6 (9.6)	11.6 (9.2)
Mean GAF score (s.d.) (*n* = 425)	59.2 (13.1)	59.0 (13.6)	59.9 (12.5)
Diagnosis, *n* (%) (*n* = 423; 245 males, 178 females)			
Schizophrenia	299 (71)	191	108
Schizo-affective disorder	56 (13)	19	37
Bipolar affective disorder	52 (12)	28	24
Depression	11 (3)	5	6
Delusional disorder	5 (1)	2	3

s.d., Standard deviation; PANSS, Positive And Negative Syndrome Scale; MADRS, Montgomery–Åsberg Depression Rating Scale; GAF, Global Assessment of Functioning.

Only 6% (*n* = 25) of the study population were not treated with antipsychotic medication. Of the participants, 30% (*n* = 127) were prescribed clozapine and 16% (*n* = 70) olanzapine. Of the participants, 75% (*n* = 321) were treated with second-generation (atypical) antipsychotics (including clozapine and olanzapine), 16% (*n* = 71) were treated with first-generation antipsychotics and 8% (*n* = 36) were treated with a combination of antipsychotics.

### Cardiovascular risk factors (see Table 3)

Of the subjects, 48% (192/398) were obese (BMI > 30 kg/m^2^). Female patients had significantly higher BMIs than males [*t* = 2.329, degrees of freedom (df) = 426, *p* < 0.020].

**Table 3.** Cardiovascular risk factors and prevalence rates by gender

Risk factor (cut-off)	*n*	Abnormal results, % (95% CI)	All subjects: mean (s.d.) (range)	Male: Mean (s.d.)/% abnormal	Female: mean (s.d.)/% abnormal
Waist circumference, cm	396	83 (78.2–86.9)	106.3 (17.1) (66–164)	106.2 (18.0)/74	107.0 (17.4)/95
Weight, kg	405		90.5 (21.1) (45–165)	93.2 (21.1)	86.9 (20.7)
Body mass index, kg/m^2^ (> 30 kg/m^2^)	398	50 (44–55)	31.0 (7.4) (14.5–58.3)	29.6 (6.3)/42	32.8 (7.7)/61
Fasting glucose, mmol/l (≥5.6 mmol/l)	322	31 (27.7–38.4)	5.9 (2.3) (3.1–26.7)	5.9 (2.3)/44	6.0 (3.3)/35
HbA1c, % (≥42 mmol/mol/[≥6.0%])	321	29 (24.9–35.6)	40.5 (8.2) (19–96)/[6.0% (1.3) (3.9–14.9)]	40.5 (7.6)/[5.9% (1.0)]/30% abnormal	40.5 (9.0)/[6.0% (1.5)]/27% abnormal
Serum triglycerides, mmol/l (≥1.7 mmol/l)	320	49 (44.0–55.7)	2.1 (1.7) (0.3–18.6)	2.3 (2.0)/56	1.7 (1.0)/39
HDL-cholesterol, mmol/l (<1.03 or 1.29 mmol/l)	322	47 (40.8–51.9)	1.2 (0.4) (0.3–2.8)	1.1 (0.3)/46	1.4 (0.4)/47
LDL-cholesterol, mmol/l (≥3.0 mmol/l)	303	51 (45.2–56.4)	3.0 (0.1) (0.3–6.3)	3.0 (1.0)/50	3.1 (1.0)/52
Serum cholesterol, mmol/l (≥5.0 mmol/l)	321	53 (47.1–58.5)	5.2 (1.6) (2.8–21.1)	5.2 (1.9)/57	5.2 (1.2)/43
Systolic BP, mmHg	385	31 (23.9–34.3)	123.0 (17.4) (74–190)	125.1 (16.4)/33	120.1 (18.4)/30
Diastolic BP, mmHg	384	40 (36–47.1)	82.4 (11.3) (45–115)	83.3 (10.7)/43	81.3 (12.0)/36

CI, Confidence interval; s.d., standard deviation; HbA1c, glycated haemoglobin; HDL, high-density lipoprotein; LDL, low-density lipoprotein; BP, blood pressure.

In all, 83% had abdominal obesity, with 95% (160/169) of females meeting the IDF criterion for central obesity, significantly higher than males (74%, *n* = 167/227) (*χ*^2^ = 29.994, *p* < 0.0001). Of the participants, 89% (305/342) had evidence of dyslipidaemia, defined as the presence of at least one abnormal lipid parameter (high total cholesterol, low high-density lipoprotein-cholesterol, high triglyceride) (*n* = 233) or treatment with lipid-lowering drugs (*n* = 72). Sixty-six people out of the 330 (20%) for whom we had data regarding either previous diagnosis of diabetes mellitus (*n* = 38) and/or had relevant blood results (*n* = 28) met the criteria for type 2 diabetes mellitus. An additional 85/286 (30%) had evidence of glucose dysregulation on their blood tests. Hypertension was present in 214/398 (54%), of whom 62 were on anti-hypertensive therapy. Nearly half (49%, *n* = 195/398) were hypertensive on measurement. Of the subjects, 62% (268/432) smoked tobacco, smoking an average of 18.2 (s.d. = 11.6) cigarettes per day.

There was no significant difference between mean BMI (*p* = 0.241) and waist circumference (*p* = 0.437) when stratified by geographical location (by borough).

The prescription of either olanzapine or clozapine (*n* = 191) (which are the antipsychotic medications traditionally associated with the highest risk of metabolic dysfunction) was not associated with an increased rate of obesity (*n* = 80, 42%) compared with those not prescribed either dibenzodiazepine (*n* = 113, 54%) (*χ*^2^ = 1.270, df = 1, *p* = 0.153). When clozapine alone (*n* = 117) was compared with other antipsychotics (excluding olanzapine), no excess in obesity was found on clozapine (47%, *n* = 55) *versus* other antipsychotics (52%, *n* = 99) (*χ*^2^ = 0.675, *p* = 0.241). Also, there was no excess of other cardiometabolic risk factors [dyslipidaemia (*p* = 0.418), hypertension (*p* = 0.546) and diabetes (*p* = 0.463)].

### Ethnicity

Those of white ethnicity had larger mean waist circumferences (mean = 108.5 cm, s.d. = 17.0) than those of black African or Caribbean ethnicity (mean = 104.4 cm, s.d. = 16.9) (*t* = 2.225, df = 345, *p* = 0.027). White patients had higher serum triglycerides than black African or Caribbean patients (mean difference = 0.89, *p* < 0.003). However, there were no significant differences in overall rates of dyslipidaemia (*p* = 0.545), hypertension (*p* = 0.101), diabetes (*p* = 0.298) and obesity (BMI > 30 kg/m^2^) (*p* = 0.140). By gender, females of black African or Caribbean ethnicity had significantly higher rates of obesity (*n* = 28/37) as measured by BMI than females of white ethnicity (*n* = 35/63) (*χ*^2^ = 4.048, *p* = 0.035).

### MetS

Of the 450 participants, there were 308 patients with complete laboratory and clinical measures to allow MetS status to be confirmed. In this group, the prevalence of IDF MetS was 56.8% (*n* = 175/308). The prevalence of the MetS as defined by the ATP-III criteria was 56.2% (*n* = 173/308). Those with IDF MetS were older (mean age = 44.68 years, s.d. = 9.8) than those without IDF MetS (42.12 years, s.d. = 10.4) (*t* = −2.28, *p* = 0.028). There was no difference in duration of illness (*p* = 0.358), or the use of clozapine (*p* = 0.282) or olanzapine (*p* = 0.303) in those with IDF MetS compared with those without. White patients had higher rates of IDF MetS (*n* = 114/171) than those of black African or Caribbean ethnicity (*n* = 47/101) (*χ*^2^ = 10.654, *p* = 0.001), with this most pronounced in white males who had significantly higher rates of IDF MetS (*n* = 66/96) than males of black African or Caribbean heritage (*n* = 29/63) (*χ*^2^ = 8.143, *p* = 0.004).

Of the 13 individuals who were not prescribed antipsychotics and who had complete measures to allow for MetS status to be confirmed, eight met the criteria for IDF MetS.

### Psychopathology and cardiometabolic risk factors (see Table 4)

A consistent relationship was found between cardiometabolic risk factors and functional impairment as measured by GAF scores. Degree of psychosis as measured by the PANSS and subscale scores was not significantly associated with any individual cardiometabolic risk factor. The only individual cardiometabolic risk factor associated with depressive symptomatology (MADRS) was obesity (*p* = 0.032)

**Table 4.** Comparison of cardiovascular risk factors with clinical characteristics

	PANSS total	*t* test/*p*	PANSS positive symptom scale score	*t* test/*p*	PANSS negative symptom scale score	*t* test/*p*	MADRS	*t* test/*p*	GAF	*t* test/*p*
Obesity										
Yes	51.6 (13.3)	−0.922/0.357	11.8 (4.8)	−0.102/0.919	13.2 (5.2)	−1.419/0.157	12.2 (9.9)	−2.151/0.032*	56.9 (12.3)	2.896/0.004*
No	50.3 (13.9)		11.7 (5.1)		12.5 (4.6)		10.1 (9.2)		60.7 (13.5)	
Diabetes										
Yes	51.9 (14.9)	−0.454/0.650	11.7 (5.0)	0.174/0.862	13.5 (5.5)	−1.107/0.269	9.9 (7.9)	0.912/0.362	56.7 (12.7)	1.653/0.099
No	51.0 (13.8)		11.8 (4.9)		12.8 (4.8)		11.1 (9.8)		59.5 (13.0)	
Hypertension										
Yes	51.0 (14.4)	0.152/0.879	11.5 (5.0)	1.224/0.222	12.9 (5.1)	−0.523/0.601	11.3 (9.7)	−0.741/0.459	57.5 (12.7)	−2.788/0.006*
No	51.2 (13.6)		12.1 (5.1)		12.7 (4.8)		10.5 (9.4)		61.2 (13.0)	
Dyslipidaemia										
Yes	51.4 (14.5)	−0.628/0.531	11.8 (5.0)	0.730/0.466	13.0 (4.9)	−1.258/0.210	11.3 (9.6)	−1.308/0.192	58.4 (12.7)	1.767/0.078
No	50.1 (11.6)		12.4 (5.1)		12.0 (4.2)		9.3 (8.6)		62.0 (14.4)	

Data are given as mean (standard deviation).

PANSS, Positive And Negative Syndrome Scale; MADRS, Montgomery–Åsberg Depression Rating Scale; GAF, Global Assessment of Functioning.

* *p* < 0.05.

There was no relationship between duration of psychotic illness and any of obesity (BMI > 30 kg/m^2^) (*t* = 0.410, df = 329, *p* = 0.682), hypertension (*t* = −1.346, *p* = 0.179), dyslipidaemia (*t* = −0.144, *p* = 0.886) or presence of diabetes mellitus (*t* = −0.992, df = 251, *p* = 0.322).

### Drug and alcohol use

Of the respondents, 28% (*n* = 68/291) had hazardous/harmful alcohol use (AUDIT score ≥ 8), while the alcohol intake of a further 4% (*n* = 12/291) indicated the need for diagnostic evaluation for alcohol dependence syndrome (AUDIT score ≥ 20).

Of the respondents, 18% (*n* = 76/422) were current cannabis users, with 52 people identified through the Timeline Follow-Back procedure and a further 24 identified through a cannabinoid-positive urinary drug screen.

#### Other substance use

Of the participants, 3% (*n* = 13/439) were cocaine or amphetamine users, 1.4% (*n* = 6) reported ongoing crack cocaine use and less than 1% (*n* = 3) reported using ecstasy.

### Lifestyle measures

Of the participants, 23% (102/435) had a high saturated fat intake score on the DINE, 29.7% (129/435) had a medium fat intake, while 47% (204/435) had a low saturated fat intake; 51% of the participants (*n* = 210) had a low fibre intake, with only 25% (*n* = 102) reporting a high fibre intake.

Of the participants, 44% (*n* = 196) were engaging in low-intensity physical activity, 44% (*n* = 200) engaged in moderate-intensity physical activity, while 12% (*n* = 54) participated in high-intensity physical activity. Engaging in high levels of physical activity was associated with lower rates of obesity (*χ*^2^ = 20.944, *p* < 0.0001) and dyslipidaemia (*χ*^2^ = 17.537, *p* = 0.001) compared with low-intensity physical activity. Further, lower rates of the MetS were found in those with high-intensity physical activity (*n* = 15/41), compared with those engaging in low-intensity physical activity (*n* = 160/267) (*χ*^2^ = 7.891, *p* = 0.004).

## Discussion

This cohort of individuals with established psychosis had a high prevalence of both individual cardiometabolic risk factors and of the MetS. Rates of central obesity were alarmingly high at 83%, with almost all women (95%) exceeding the IDF threshold. Half the sample were obese, half had hypertriglyceridaemia and 54% were hypertensive. Of the study population, 50% had evidence of glucose dysregulation, including 20% of the sample with type 2 diabetes, which is much higher than the rate in the general UK population (International Diabetes Federation, 2013). This excess is amplified when an aged-matched comparison is used; the expected prevalence of diabetes in UK residents with an age range from 35 to 54 years would be 9.4% for men and 6.6% for women (Craig & Mindell, 2011).

Of our participants, 56.8% met criteria for the MetS, greatly exceeding the international figure of 34% described in a recent meta-analysis of the MetS in schizophrenia (Mitchell *et al.* 2013). This meta-analysis found a lack of homogeneity in the distribution of MetS prevalence across individual studies. Similar rates of cardiometabolic risk to what we found have been identified in a large Australian prevalence survey in psychosis, where rates of abdominal obesity greater than 80% were found, as well as comparable rates of the MetS, dyslipidaemia and hypertension (Galletly *et al.* 2012). Our prevalence rates thus place this UK population at the higher end globally for cardiometabolic risk factors in psychosis. Given that UK general population studies have identified rates of the MetS of 25–34%, the almost doubled rate in psychosis is striking (Khunti *et al.* 2010; Langan *et al.* 2012) and behoves us to investigate the antecedents and remedies. Consistent with general population studies, but in contrast to some cross-sectional studies in psychosis, we found no gender difference in the rates of the MetS (Ford, 2005), although we did find higher rates of obesity (as defined by BMI) in females (61% *v.* 42%) (McEvoy *et al.* 2005; Bobes *et al.* 2007). There were ethnic differences in rates of the MetS, with those of white ethnicity showing higher rates compared with those of black African or Caribbean ethnicity, in keeping with previous work (McEvoy *et al.* 2005; Keenan *et al.* 2013), results which have not been consistently demonstrated in the general population (Keenan *et al.* 2013).

Lifestyle choices amplify the potential consequences of abnormal metabolic parameters in this population: 62% were smokers, hugely in excess of the current smoking rates in the general UK population of 20% (Health & Social Care Information Centre, 2013). A recent 11-year follow-up of 517 people with schizophrenia showed an overall standardized mortality ratio of 2.80, smoking being a very strong predictor of death (relative risk of 4.66) (Dickerson *et al.* 2014). Smoking not only increases the risk of early death, but also complicates treatment, in that the hydrocarbons in cigarette smoke accelerate the metabolism of dibenzodiazepines, clozapine and olanzapine (Rostami-Hodjegan *et al.* 2004). Until recently, clinicians have shown little interest in supporting smoking cessation in psychosis patients. However, findings from a systematic review have demonstrated that people with schizophrenia want to stop smoking and that it is possible to help them to do so (Banham & Gilbody, 2010) and it is logical that this should now be a priority public health measure.

Lack of exercise was common among the study population, with only 12% of individuals participating in high-intensity physical activity, consistent with previous reports (Faulkner *et al.* 2006), with high-intensity physical activity associated with lower rates of obesity and dyslipidaemia. As exercise is linked to greater longevity in the general population (Paffenbarger *et al.* 1993; Manson *et al.* 2002), it is likely that greater emphasis on engaging in physical activity among those with severe mental illnesses (SMI) would help reduce mortality risk (Vancampfort *et al.* 2010). This will require addressing the individual barriers to regular physical activity and promoting it within the structure of community treatment programmes (Vancampfort *et al.* 2012) but will also entail addressing wider organizational barriers to regular physical activity and other health promotion programmes (O'Brien *et al.* 2014).

We did not identify any associations between individuals’ psychopathology scores on PANSS and their cardiometabolic risk. However, we did find associations between increased functional impairment and obesity, diabetes and hypertension and between higher depression scores and obesity. Of note, in the US Clinical Antipsychotic Trials of Intervention Effectiveness (CATIE) study, the PANSS mean total score of 75.9 was higher than in IMPaCT (51.2) – with lower NCEP ATP-III MetS rates of 36% in CATIE compared with the ATP-III MetS rate of 56% in IMPaCT, meaning that our higher rates of cardiometabolic risk are not a result of illness severity (Meyer *et al.* 2005).

The rate of the MetS in the BPAD subgroup was 66%, which is higher than previously reported rates of 22–30% (Garcia-Portilla *et al.* 2008; van Winkel *et al.* 2008). This may relate more to service bias than diagnostic factors. Patients with bipolar disorder requiring ongoing secondary care management in the UK may have a greater clinical need than those managed in primary care and as we have demonstrated an effect of global function on cardiometabolic risk, this is one potential explanation, although the numbers were too small to test this hypothesis.

Despite the mean duration of illness being 15.7 years, we found no effect of duration of illness on the prevalence of the MetS, in contrast to the meta-analysis conducted by Mitchell *et al.* (2013). Even though our study excluded, for pragmatic reasons, those people registered with early intervention services, which manage younger people for up to 3 years from first presentation, we would have expected to find an effect.

A high proportion of this sample was prescribed dibenzodiazepines, which are associated with early emergence of cardiometabolic risk factors (Howes *et al.* 2004; Nielsen *et al.* 2010). However, we found no differences in cardiac risk factors between those on clozapine or olanzapine and those not. One potential explanation is that the cardiometabolic effect plateaus and becomes more evenly distributed over a more prolonged course of illness. However, as we do not have access to treatment histories, it may also be that this homogeneity across medications reflects the cumulative effects of medication changes over the years. This lack of difference is especially interesting in light of the evidence that patients with schizophrenia treated with clozapine have the least reduction in life expectancy of all patients with schizophrenia (Tiihonen *et al.* 2009; Hayes *et al.* 2014).

Strengths of this study include a sample of randomly selected individuals with psychosis from diverse ethnic backgrounds and the mix of urban and rural locations adding to the generalizability of the study findings. Limitations of the study include the cross-sectional design, and the lack of a control group. We have not adjusted for the effect of socio-economic deprivation. The sample was recruited from across England, but predominantly from Greater London, including some boroughs with high levels of deprivation, though we identified no difference in metabolic variables by borough. A recent Scottish paper noted an excess of mortality in SMI linked to deprivation, although this effect was not evident for deaths due to cerebrovascular disease or CVD (Langan Martin *et al.* 2014). The missing data from metabolic measures and clinical assessment scales may have introduced a selection bias into the study, but it is not obvious what effect such a bias would result in. Measures such as duration of illness were self-reported and thus are non-validated. We have no data on duration of antipsychotic treatment and previous antipsychotic treatments used over the longitudinal course of an individual's illness, both of which are confounding factors that could potentially make an impact on an individual's cardiometabolic risk factors. The exclusion of FEP patients from this study recognizes the different CVD profiles in the early stages of psychosis. Data on the evolution of CVD risk factors from first presentation are being gathered as part of a different project within IMPaCT. To have included FEP data within this study would have underestimated the extent of the problem which patients with established psychosis, their carers and their clinicians face.

Because of the design of the study, individuals with established psychosis cared for by their general practitioners were not included in the study population. This group is estimated to make up 30% of all patients with SMI in the UK (Reilly *et al.* 2012). Our study population, compared with this primary care-only population, may be functionally or symptomatically more impaired; and antipsychotic prescribing patterns may be different – for instance, clozapine is rarely prescribed in primary care. Thus the study's findings may not necessarily generalize to those patients living in the community who share these same diagnoses but who are not managed in secondary care.

## Conclusion

The results from this study are alarming and draw attention to the huge rates of modifiable cardiometabolic risk factors in people with established psychosis in the UK. The prevalence of the MetS in people with established psychosis in this UK sample is 56.8%. The main defining measure of the MetS, waist circumference, exceeded the IDF diagnostic cut-off in 83% of our patients, and in 95% of female patients. Although central obesity is known to be the best predictor of morbidity and mortality (Zimmet *et al.* 2001), waist circumference has not to date been routinely measured in people with psychosis, although it has now been included in the latest National Institute of Health and Clinical Excellence (2014) guidelines for schizophrenia. Practice in in-patient psychiatric services has improved with the advent of incentivization and of shared guidelines for CVD risk management in psychosis such as the Lester Cardiometabolic Health Resource (Lester *et al.* 2014). However, serious shortcomings remain in the management of physical health care in community-based patients, as evidenced by the National Audit of Schizophrenia findings (NAS, 2012; Crawford *et al.* 2014). Nevertheless, more tailored work is needed if we wish to improve life expectancy in psychosis, with these findings reinforcing the need for routine monitoring of waist circumference in psychosis and assertive management of cardiovascular risk in people with psychosis.
